# *Salmonella* identified in pigs in Kenya and Malawi reveals the potential for zoonotic transmission in emerging pork markets

**DOI:** 10.1371/journal.pntd.0008796

**Published:** 2020-11-24

**Authors:** Catherine N. Wilson, Caisey V. Pulford, James Akoko, Blanca Perez Sepulveda, Alexander V. Predeus, Jessica Bevington, Patricia Duncan, Neil Hall, Paul Wigley, Nicholas Feasey, Gina Pinchbeck, Jay C. D. Hinton, Melita A. Gordon, Eric M. Fèvre

**Affiliations:** 1 Institute of Infection, Veterinary and Ecological Sciences, University of Liverpool, Liverpool, United Kingdom; 2 Malawi-Liverpool Wellcome Trust Clinical Research Programme, Blantyre, Malawi; 3 International Livestock Research Institute, Nairobi, Kenya; 4 Maseno University, Kisumu, Kenya; 5 Ministry of Agriculture, Food Security, Irrigation and Water Development, Malawi Government; 6 Earlham Institute, Norwich, United Kingdom; 7 Department of Clinical Sciences, Liverpool School of Tropical Medicine, Liverpool, United Kingdom; Creighton University, UNITED STATES

## Abstract

*Salmonella* is a major cause of foodborne disease globally. Pigs can carry and shed non-typhoidal *Salmonella* (NTS) asymptomatically, representing a significant reservoir for these pathogens. To investigate *Salmonella* carriage by African domestic pigs, faecal and mesenteric lymph node samples were taken at slaughter in Nairobi, Busia (Kenya) and Chikwawa (Malawi) between October 2016 and May 2017. Selective culture, antisera testing and whole genome sequencing were performed on samples from 647 pigs; the prevalence of NTS carriage was 12.7% in Busia, 9.1% in Nairobi and 24.6% in Chikwawa. Two isolates of *S*. Typhimurium ST313 were isolated, but were more closely related to ST313 isolates associated with gastroenteritis in the UK than bloodstream infection in Africa. The discovery of porcine NTS carriage in Kenya and Malawi reveals potential for zoonotic transmission of diarrhoeal strains to humans in these countries, but not for transmission of clades specifically associated with invasive NTS disease in Africa.

## Author summary

Healthy humans infected with non-typhoidal *Salmonella* (NTS) typically suffer from diarrhoeal disease which resolves without treatment. However, NTS infection of patients with an impaired immune system can lead to either bloodstream infection or infection in another part of the body; so-called ‘invasive’ NTS infection. Over the last twenty years, NTS have been the most prevalent bacteria to be isolated from human blood in sub-Saharan Africa.

It is well known that pigs are able to carry a wide range of different NTS serovars without showing signs of disease themselves. Carrier pigs may be able to intermittently shed NTS in their faeces which may have the potential to cause disease in humans. If good hygiene protocols are not followed during the porcine slaughter process, pork intended for human consumption may become contaminated with NTS. In the next few years, pork consumption is projected to increase dramatically in sub-Saharan Africa. The degree of asymptomatic carriage of NTS by pigs in this region has never been investigated in detail.

In this study, we report that pigs in sub-Saharan Africa are able to carry a wide variety of non-typhoidal *Salmonella* serovars, such as *S*. Typhimurium, that have the potential to cause diarrhoeal disease in humans. No clades of NTS which have previously been associated with invasive NTS disease in sub-Saharan Africa were detected.

## Introduction

Infection with non-typhoidal *Salmonella* (NTS) in healthy humans is typically associated with self-limiting enterocolitis, but in immunocompromised patients can lead to bloodstream or focal metastatic infections [1]. However over the past two decades, NTS have been the most prevalent bacteria to be isolated from human blood in sub-Saharan Africa (sSA) [2–5]. The main risk factors for invasive NTS (iNTS) disease are HIV [6], malaria [7], and malnutrition [8]. The emergence of iNTS disease has been associated with specific, multidrug resistant clades of *Salmonella* [9,10]. Despite an increasing amount of evidence suggesting human adaptation [11,12], the reservoir for these novel lineages has not been established.

Pigs act as a reservoir for NTS as they can carry a diverse range of *Salmonella* serovars asymptomatically in the tonsils, intestine and mesenteric lymph node (MLN) tissue [13]. Carrier pigs intermittently shed potentially pathogenic *Salmonella* bacteria via faeces, and pork may become contaminated during slaughter processes if proper procedures and hygiene are not observed, for example, incorrect hanging of carcasses for evisceration or contact between the meat and a soiled floor can transmit *Salmonella* [14]. Extensive work has been undertaken to investigate *Salmonella* carriage and excretion by pigs in Europe and the USA, where the most common *Salmonella* serovars isolated are *S*. Derby, *S*. Enteritidis and *S*. Typhimurium [15–19]. These serovars frequently cause human infection, accounting for 43.6% of cases of gastroenteritis-associated salmonellosis in southern Europe [20]. Consequently, porcine carriers of *Salmonella* are considered to pose a threat to public health.

Currently the published data from sSA countries is limited [21]. Pork consumption and supply in Kenya is estimated to rise by 268% between 2010–2050 [22] a trend that is predicted to be replicated across sSA [23]. Pigs in many rural areas of Kenya and Malawi are free-roaming with access to human faeces in areas where open-defecation occurs, and often defecate in close proximity to human domestic environments, which may facilitate zoonotic transmission [24]. To investigate the prevalence and diversity of NTS in pigs in sSA, we isolated *Salmonella* from the faeces and MLN of pigs at slaughter in Kenya and Malawi.

## Materials and methods

### Location and sampling

Pigs included in this study were those brought for slaughter on the day of sample collection at designated slaughterhouses in three study sites: Busia (Kenya), Nairobi (Kenya) and Chikwawa (Malawi). Samples were collected between October 2016 and May 2017. See S1 Table for more details.

Faecal and MLN samples were taken from pigs *post mortem*. Between 1 and 25g faeces were taken manually directly from each pig rectum. Once the entire gastrointestinal tract had been removed during meat processing, between 1-4g of MLN tissue was excised using a sterile scalpel. At least five individual MLN were sampled per pig. Approximately three of the lymph node samples were taken from the mesentery of the ileum and jejunum, and two samples were taken from the colonic mesentery, and samples from each animal were pooled (total 1 to 4g). Slaughtermen in Chikwawa were extensively trained in the sampling methodology prior to commencing the study. Samples were processed in the laboratory within four hours of collection.

Additional metadata were collected on paper (Malawi) and electronically (Kenya) using a ‘Field Information Support Tool’ developed from a Case Report Form by the Kestrel Technology Group (Kestrel Technology Group, LLC) on a Nexus 5 Android device. This questionnaire included name of the village where the pig was reared, previous antibiotic treatment, age, sex, breed of pig and method of transport of the pig to slaughterhouse. The GPS location of each of the slaughterhouses in Kenya and butcheries in Malawi was recorded.

### Microbiological methods (Fig 1)

NTS were isolated by culture using standard procedures (International Organisation for Standardisation (ISO 6579:2002). The exterior of the surface of each of the MLN samples was placed briefly into a flame to remove any residual exterior contamination. 1g of MLN and 1g of faeces from each pig were placed into separate stomacher bags containing 9ml 2% buffered peptone water. Samples were homogenized and each sample was incubated for 18hours (h) at 37°C in air. Following pre-enrichment, 0.1ml of each sample was placed into a sterile bijou containing 9.9ml Rappaport-Vassiliadis broth, and were incubated for 24h at 42°C. Each enriched sample was inoculated onto both Brilliant Green agar and Harlequin ABC *Salmonella* plates. Following 24h incubation, positive colonies were inoculated onto nutrient agar plates and incubated for a further 24h at 37°C prior to antisera agglutination and antimicrobial susceptibility testing. *Salmonella* agglutination was carried out using *Salmonella* antisera (Polyvalent O-antigen and H Phase 1 and 2 or H Phase 2 antigens). Isolates which showed positive agglutination with Poly O Antigen and either H Phase 1 and 2, or H Phase 2 antigens were submitted for Whole Genome Sequencing (WGS) as presumptive salmonellae.

**Fig 1:**
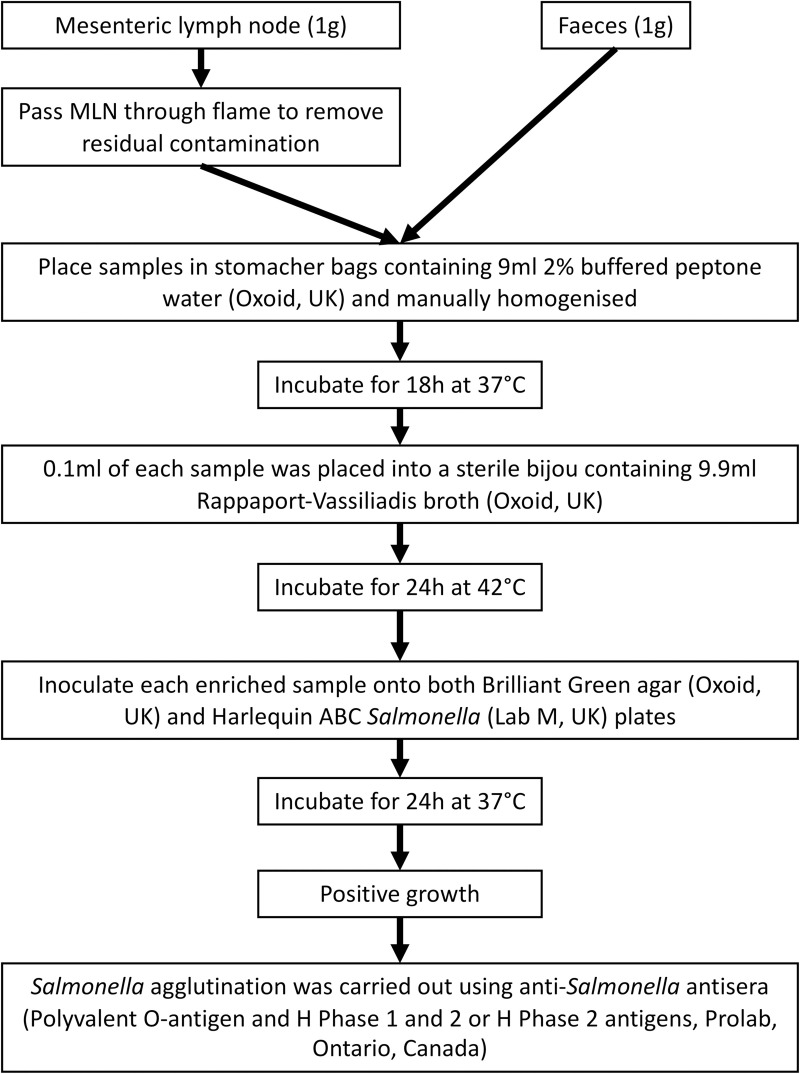
Microbiological Methods. An outline of the microbiological methods followed in the laboratory to undertake sample processing.

### Whole genome sequencing

Presumptive *Salmonella* samples were submitted for WGS to the Earlham Institute, Norwich as part of the 10,000 *Salmonella* Genomes Project (https://10k-salmonella-genomes.com/) [25]. An individual *Salmonella* colony was inoculated into each 0.7ml FluidX 2D tri-coded jacket tube (FluidX Ltd, UK) containing 100μl 2% buffered peptone water solution (Oxoid), and incubated at 37°C for 24h. The FluidX 2D Tubes were then placed in a 95°C oven for 20 minutes to inactivate the isolates. DNA extraction was carried out by the Earlham Institute and library preparation was performed using a modified Illumina Nextera XT DNA Library Prep Kit (Illumina, FC-131-1096). Illumina short-read sequencing was carried out on these samples to achieve 150bp paired-end reads using the HiSeq 4000. Sequencing was multiplexed using 768 unique barcode combinations per sequencing lane. The insert size was approximately 180bp, and the median depth of coverage was 30x.

### Quality control and read trimming

Paired-end reads were subjected to stringent quality checks using FastQC v0.11.5 (www.bioinformatics.babraham.ac.uk/projects/fastqc/) and MultiQC v1.0 (http://multiqc.info). Potentially contaminated sequences were detected using Kraken v0.10.5-beta [26] against the MiniKraken 8gb database, using a *Salmonella* abundance cut-off of 70%. The paired-end reads were adapter-trimmed using palindromic Trimmomatic v0.36 [27], and quality trimmed using SEQTK v1.3-r106 (https://github.com/lh3/seqtk).

### Assembly and annotation

Unicycler v0.3.0b [28] was used to produce high quality genome assemblies which were assessed using QUAST. Genomes that exceeded the quality control metrics defined by Enterobase [29] were designated as high quality, and used for subsequent analysis. Annotation was performed using Prokka v1.12 [30] against a custom-made database of *Salmonella-*specific genes.

### *In silico* typing

*In vitro Salmonella* serotyping was confirmed using the *Salmonella in Silico* Typing Resource (SISTR)[31]. The strains were also assigned a Multi Locus Sequence Type (MLST) using the software tool MLST v2.10 [32] based on the conservation of seven housekeeping genes.

### Core gene-based phylogenetics

**Fig 2:**
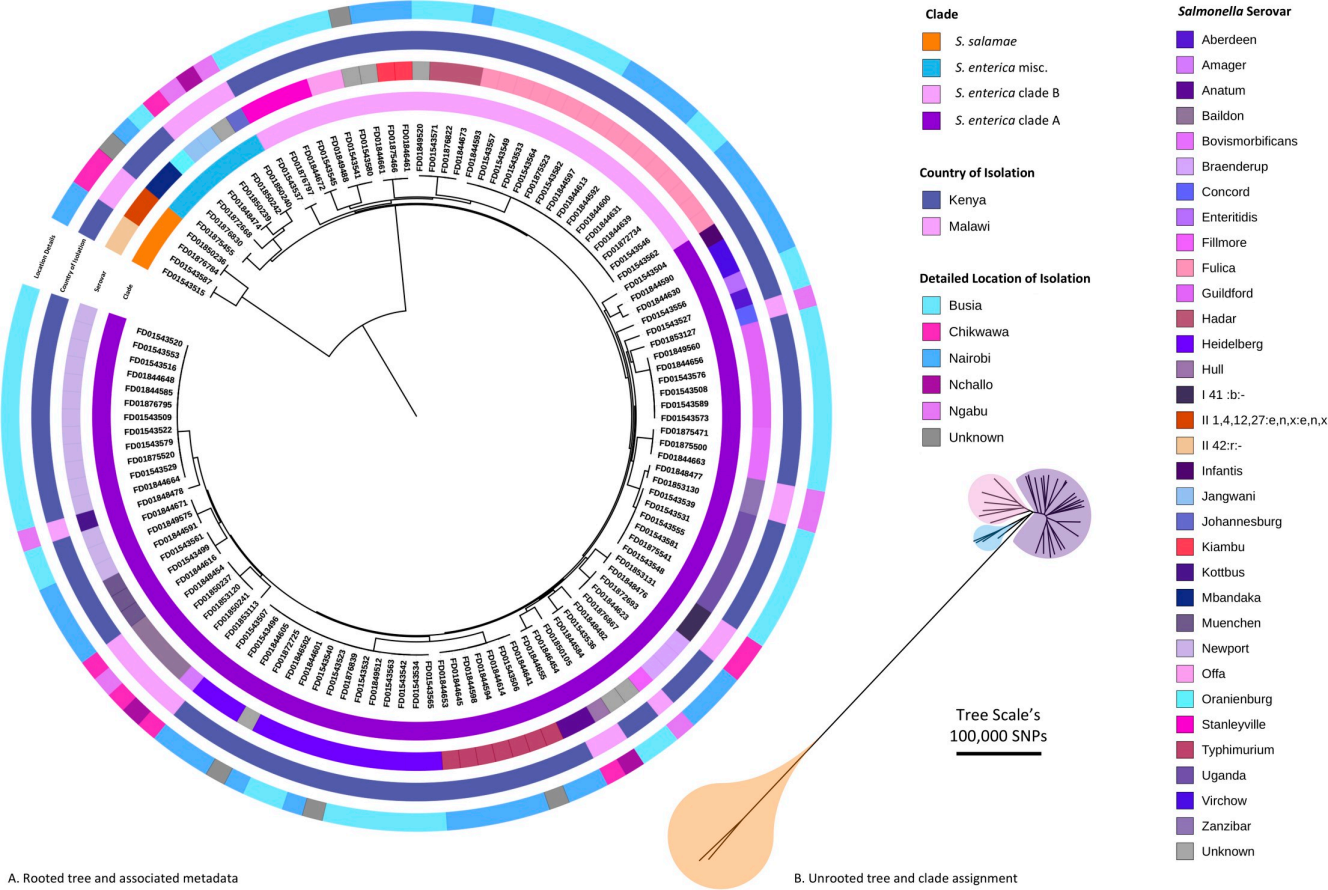
The diversity of pig-derived *Salmonella* identified in Kenya and Malawi. A maximum likelihood phylogenetic tree based on core gene SNPs. The tree was rooted at the inferred position of the outgroup *S*. *bongori*. B. Maximum likelihood phylogenetic tree (unrooted). Note the colours refer to clade designation. Both visualised using ITOL (https://itol.embl.de).

To investigate the relationship between the diverse set of 121 high-quality pig-derived *Salmonella* genomes a maximum likelihood phylogeny was inferred from a core gene SNP alignment. Core genes were defined as present in at least 99% of genomes. Roary v3.11 [33] and SNP sites v2.3.3 software were used to generate the alignment which comprised of 3,010 core genes and 208,657 sites. The maximum likelihood tree was built using RAxML-NG v0.4.1 BETA [34], with the general time reversible (GTR) model and gamma distribution for site-specific variation and 100 bootstrap replicates to assess support. To infer the position of the root, the phylogeny was rebuilt using *S*. *bongori* as an outgroup. The finished phylogeny (Fig 2) was then rooted according to the inferred position using the Interactive Tree Of Life (iTOL) v4.2 [35].

### Contextualising pig-derived *S*. Typhimurium ST313 isolates

We describe below the finding of *S*. Typhimurium sequence type 313 (ST313) in some of our samples. Given the specific public health importance of this ST, and to place the pig-derived *S*. Typhimurium ST313 isolates into a global context, a phylogeny was constructed that included 207 published ST313 genomes (S2 Table)[36–40]. A single nucleotide polymorphism (SNP) alignment was inferred from 2,004 core genes using Roary v3.11 [33] and SNP sites v2.3.3 [41]. The alignment comprised of 4,999 SNP sites. The final maximum likelihood tree was built using RAxML-NG v0.4.1 BETA [34] with 100 bootstrap replicates to assess support. The relatedness of the pig-derived ST313 was visualised with iTOL v4.2 [35].

The Short Read Sequence Typing for Bacterial Pathogens (SRST2) v0.2.0 [42] software tool was used to detect the presence of plasmid and prophage sequences associated with ST313, using a custom-made database based on plasmid and prophage sequences present in the ST313 reference strain D23580 and known variants. Reporting of gene presence is based on 90% coverage against the reference sequences. For pairwise comparison, pig-derived ST313 contigs were ordered against the ST313 reference genome D23580 using ABACAS v1.3.1 [43]. A pairwise comparison file was then generated between the ordered assemblies using BLASTn with default parameters, and visualised with the Artemis Comparison Tool v10.2 [44] (S1 Fig).

### Antimicrobial resistance (AMR) testing

Genetic determinants for antimicrobial resistance were identified using Staramr v0.5.1 (https://github.com/phac-nml/staramr) against the ResFinder [45] and PointFinder [46] databases. An acquired AMR gene was considered to be present in a genome if percentage nucleotide homology was >90%. Confirmatory phenotypic antimicrobial susceptibility testing was carried out by disk diffusion on any isolates that contained antimicrobial-resistance determinants according to the European Committee on Antimicrobial Susceptibility Testing (EUCAST) guidelines [47]. Isolates were tested in duplicate for susceptibility to seven antibiotics (pefloxacin 5μg, trimethoprim/sulfamethoxazole 25μg, tetracycline 30μg, fosfomycin/glucose6phosphate 200μg, ceftriaxone 30μg, ampicillin 10μg and gentamicin 10μg) (all disks from Mast Group). Plates were incubated for 18-24h at 37°C, and the zones of inhibition were read for each disk to the nearest millimetre. According to EUCAST breakpoint tables for Enterobacteriaceae, isolates were classified as either susceptible or resistant to each antibiotic [48]. Phenotypic results were correlated with the genome-derived identification of antimicrobial resistance genes for each isolate.

### Statistical analysis

Descriptive statistics with 95% confidence intervals were used to describe the prevalence and diversity of NTS detected using Microsoft Excel version 15.31. The frequency and diversity of the antimicrobial susceptibility phenotypes and genotypes of the NTS detected were also analysed using descriptive statistics with a 95% confidence interval.

### Ethics

Ethical approval for this study was obtained from the University of Liverpool Veterinary Research Ethics Committee (Reference number VREC465), the Kenya National Commission for Science Technology and Innovation accredited International Livestock Research Institute Institutional Animal Care and Use Committee, Nairobi, Kenya (IACUC reference number 2016.19) and the College of Medicine Research Ethics Committee, Malawi (reference number P.02/17/2124).

## Results

### Descriptive epidemiology

Faeces and MLN were sampled from 647 pigs across the three study areas (Busia = 276, Nairobi = 306 and Chikwawa = 65). Isolates that showed positive agglutination using the *Salmonella* O antigen test were obtained from 259 pigs. All 259 isolates were submitted for whole genome sequencing, of which 149 were genotyped initially as being NTS. 28/149 isolates failed quality control checks, therefore 121 isolates were genotyped as being NTS. This gives an overall prevalence of NTS of 12.7% (95% confidence interval (CI); 8.75–16.6%) in Busia, 9.2% (95%CI; 5.9–12.4%) in Nairobi and 24.6% (95%CI; 14.1%-35.1%) in Chikwawa (See Table 1, S2 Fig). Several pigs were found to be carrying more than one serovar of NTS; 5.7% of pigs from Busia, 7.15% from Nairobi and 6.3% from Chikwawa (Table 1, S2 Fig).

**Table 1:** Prevalence of non-typhoidal *Salmonella* serovars.

Sampling location	Total number of NTS isolates	Number of pigs in which NTS was detected	Percentage pigs carrying NTS (%)	Number of NTS isolates detected from mesenteric lymph node samples	Number of NTS isolates detected from faecal samples	Number of pigs in which more than 1 NTS isolate was detected*	Number of pigs carrying more than 1 serovar of NTS
Busia (n = 276)	61	35	12.7 (8.7–16.6)	44	16	22	2
Nairobi (n = 306)	40	28	9.1 (5.9–12.4)	21	19	12	2
Chikwawa (n = 65)	20	16	24.6 (14.1–35.1)	18	2	9	1

* This means that more than one NTS isolate was isolated during the culture method or from the faecal and mesenteric lymph node samples from one pig.

### Serotypes and phylogenetic-relatedness of pig-derived *Salmonella*

**Fig 3:**
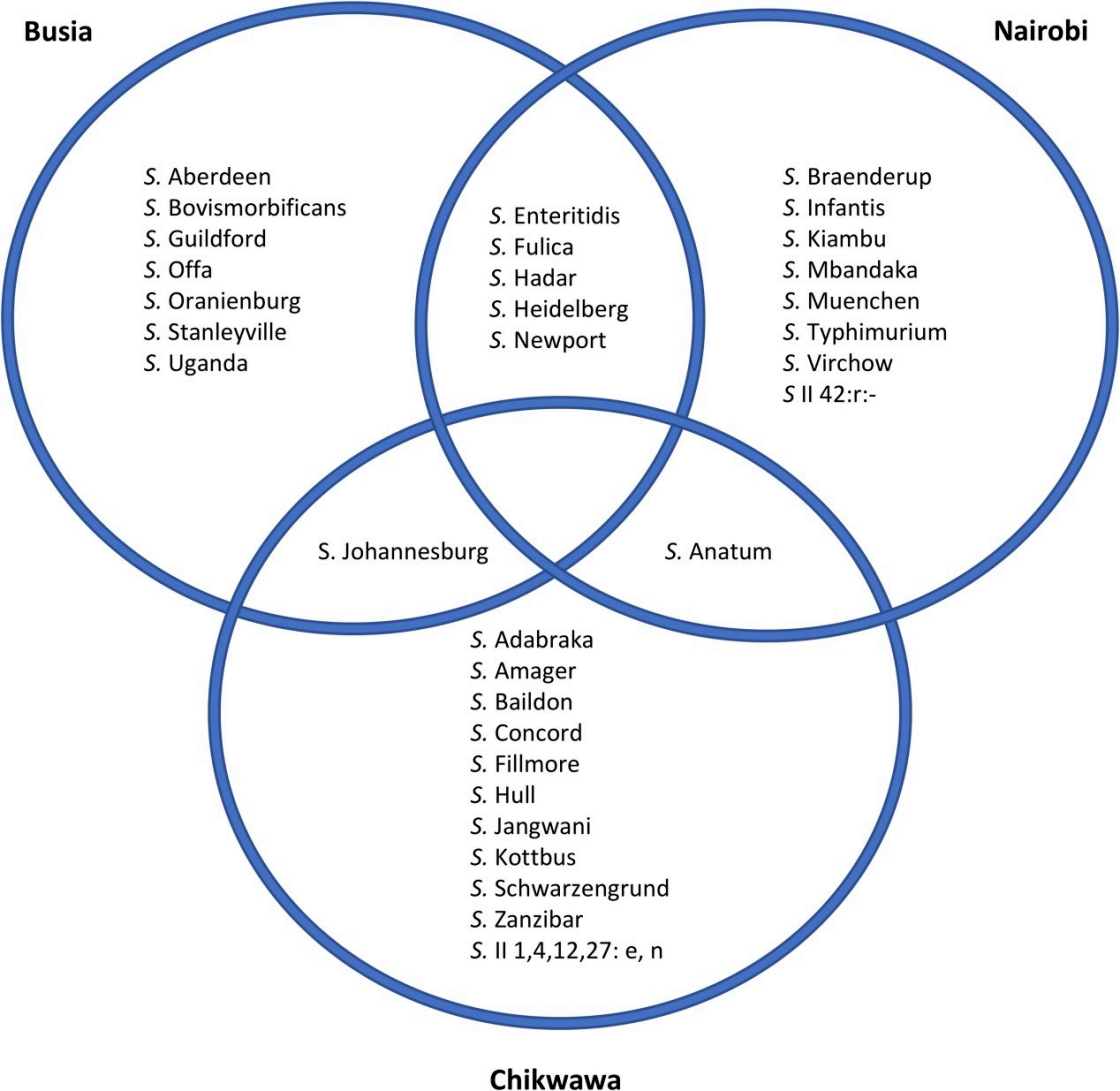
The *Salmonella* serovars detected in each of the study locations.

To visualise the diversity of *Salmonella* identified, the genomic data were used to derive serotype information from 121 isolates, and a core gene SNP-based phylogeny was constructed (Fig 2). Thirty-two different *Salmonella* serovars were identified from two sub-species, *S*. *enterica* and *S*. *salamae*. Most serovars were unique to a single sampling site, seven serovars were found in two sampling sites; Nairobi and Busia (n = 5), Nairobi and Chikwawa (n = 1) and Busia and Chikwawa (n = 1). No serovars were detected in all three study sites (Fig 3). In total 8 isolates of *S*. Typhimurium were identified; 6 isolates of *S*. Typhimurium ST19 isolated from 4 pigs, and 2 isolates of S. Typhimurium ST313 isolated from 2 pigs.

Phylogenetically, the *Salmonella* isolates belonged to four broadly defined groups, which corresponded to previously characterised *Salmonella* subspecies and clades (Fig 2, S3 Table). The largest clade (n = 80) included 66% of the isolates and 20 serovars, and is known as Clade A of *S*. *enterica* subspecies enterica [49], a grouping that contains serovars responsible for the majority of human disease. A second group of 30 *S*. *enterica* subspecies enterica isolates belonged to clade B [49], and originated from pigs in the two Kenyan study sites, 50% of which were the *S*. Fulica serovar. A third, smaller cluster of 7 *S*. *enterica* isolates did not belong to clade A or clade B. Four of these isolates were typed as *S*. *salamae*.

### Antimicrobial resistance

We identified that 28/121 (23.1%) NTS isolates carried antimicrobial resistance (AMR) genes. These include 15/40 (37.5%) isolates from Nairobi, 12/61 (19.7%) isolates from Busia, and 1/20 (5.0%) isolate from Malawi. To determine the concordance between phenotypic and genotypic characterisation in our study, we analysed the antibiotic susceptibility phenotype of 26 genotypically resistant isolates (two of the isolates were unavailable for testing) (Table 2). Phenotypically, 16/26 isolates were susceptible to all antibiotics tested, 6/26 isolates were resistant to one antibiotic, 4/26 NTS isolates were resistant to two classes of antibiotics and none of the isolates were classified as multi-drug resistant (resistant to three or more classes of antibiotics).

**Table 2:** Antimicrobial susceptibility phenotypes and genotypes in pig-derived *Salmonella*. Heat map of antimicrobial resistance determinants and resistance phenotypes linked to 28 pig derived *Salmonella* isolates. Phenotype is displayed using colour, with green representing susceptibility and red representing resistance, according to guidelines set by EUCAST [47]. Light green represents those isolates for which antimicrobial susceptibility testing was not available (2/28 isolates). The antibiotic resistance genes that were identified by staramr v0.5.1 (https://github.com/phac-nml/staramr) are displayed in white text.

	Pefloxacin	Ceftriaxone	Fosfomycin	Tetracycline	Trimethoprim-Sulfamethoxazole	Ampicillin	Gentamicin
FD01853127					dfrA14 sul2		aph**(3**'')-1b aph**(6)-**1d
FD01543571				tet(A)	dfrA14 sul2		aph**(3**'')-1b aph**(6)-**1d
FD01849520				tet(A)	dfrA14 sul2		aph**(3**'')-1b aph**(6)-**1d
FD01844591					sul2		aph**(3**'')-1b aph**(6)-**1d
FD01844594				tet(A)	sul2		aph**(3**'')-1b aph**(6)-**1d
FD01844598				tet(A)	sul2		aph**(3**'')-1b aph**(6)-**1d
FD01844614				tet(A)	sul2		aph**(3**'')-1b aph**(6)-**1d
FD01844645				tet(A)	sul2		aph**(3**'')-1b aph**(6)-**1d
FD01844653				tet(A)	sul2		aph**(3**'')-1b aph**(6)-**1d
FD01876797					sul2	blaTEM-1B	
FD01543496			fosA7				
FD01543507			fosA7				
FD01543523			fosA7				
FD01543532			fosA7				
FD01543534			fosA7				
FD01543540			fosA7				
FD01543542			fosA7				
FD01543563			fosA7				
FD01543565			fosA7				
FD01844601			fosA7				
FD01844605			fosA7				
FD01846502			fosA7				
FD01849512			fosA7				
FD01872668			fosA7				
FD01872725			fosA7				
FD01876839			fosA7				
FD01543506	gyrA (D87Y)		fosA7				
FD01844630				tet(J)			

We found that 16/121 (13.2%) isolates carried *fosA*, the genetic determinant for resistance to fosfomycin. The presence of this gene was strongly associated with *S*. Heidelberg (15/16), but all 16 isolates were phenotypically susceptible to fosfomycin (Table 2). There were 8/121 (6.6%) isolates that harboured tetracycline resistance genes *tetA* (5.8%) or *tetJ* (0.8%), however 6/7 isolates carrying *tetA* (85.7%) and the single isolate carrying *tetJ* were phenotypically susceptible to tetracycline. One isolate showed phenotypic resistance to tetracycline, despite the absence of genomic predictions of known tetracycline resistance genes. 10/121 (8.3%) isolates contained sulphonamide resistance gene *sul2*, of which three carried *dfrA14*, and all three were phenotypically resistant to cotrimoxazole. One isolate that carried *sul2* but not *dfrA14* was also phenotypically resistant to co-trimoxazole. One isolate from a pig in Nairobi carried *blaTEM-1B*. The isolate was resistant to ampicillin and sensitive to ceftriaxone on phenotypic testing. One additional isolate showed phenotypic resistance to ampicillin but did not carry any known ampicillin resistance genes. We identified 9/121 (7.4%) isolates that carried the genes *aph(3”)-1b* and *aph(6)-1d*, both of which are associated with resistance to aminoglycosides; none of these isolates demonstrated phenotypic resistance against gentamicin. One isolate from a pig in Busia had a point mutation (D87Y) in *gyrA*, associated with resistance to fluoroquinolones and this was confirmed by testing the isolate against pefloxacin (Table 2).

### *S*. Typhimurium ST313 analysis

**Fig 4:**
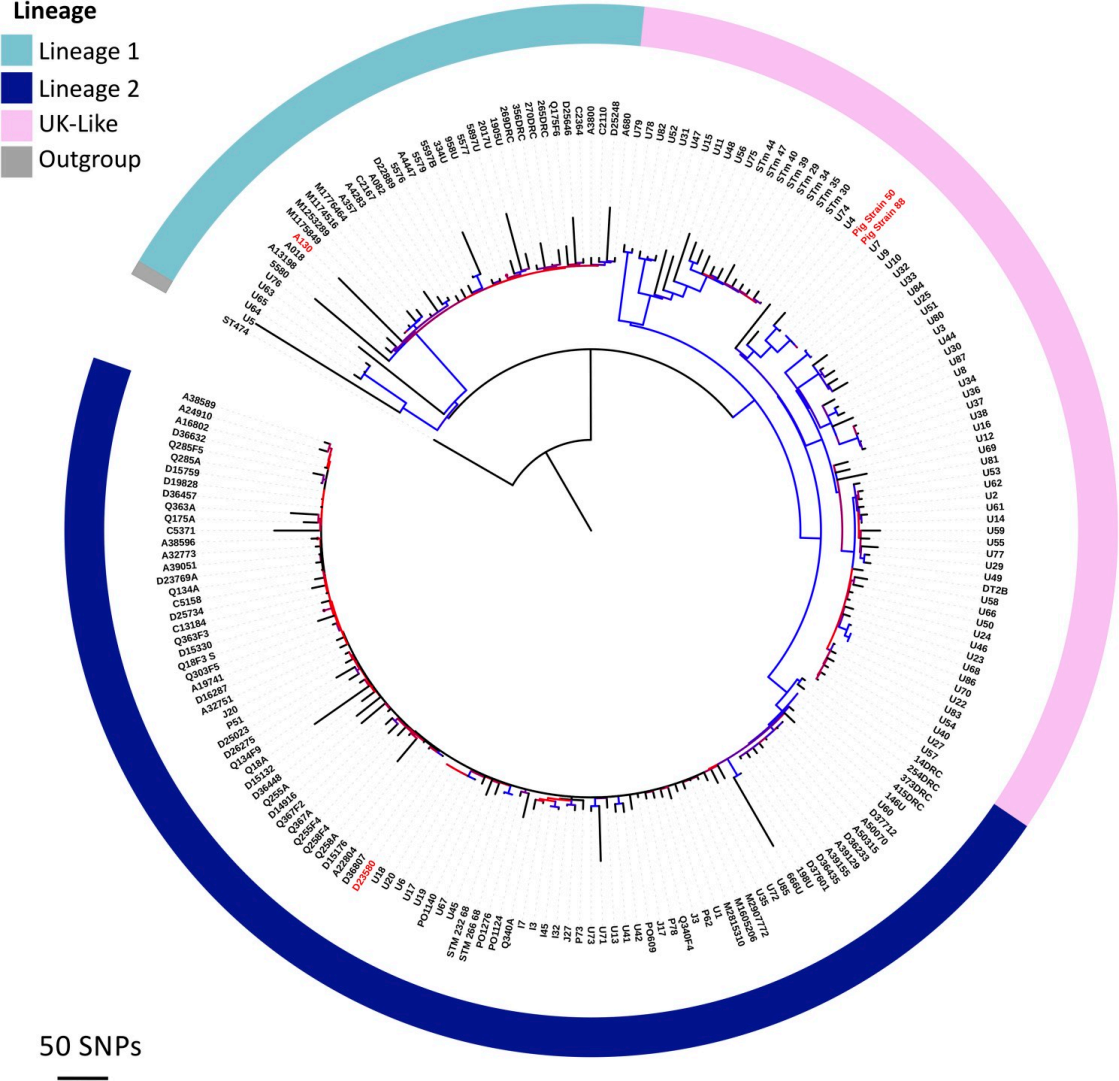
Two pig-derived *S*. Typhimurium ST313 isolates in the context of published ST313 genomes. **The** A maximum likelihood phylogenetic tree based on core genome SNPs. Phylogeny is outgroup-rooted to *S*. Typhimurium ST19 strain 4/74 [57]. Visualised using ITOL (https://itol.embl.de).

Two *S*. Typhimurium ST313 were isolated from the MLN of individual pigs slaughtered in Nairobi (FD01844610 and FD01844641), and differed by 110 core gene SNPs. Neither isolate contained AMR genes, and both were shown to be antibiotic-susceptible phenotypically (Table 2). To determine the similarity between the two ST313 isolated here and *S*. Typhimurium ST313 currently causing an epidemic of iNTS in Africa, a core gene SNP-based phylogeny of the two genomes alongside published ST313 genomes was constructed (Fig 4). The resulting phylogeny showed that both isolates were related to a diverse group of isolates associated with human gastrointestinal disease in the United Kingdom, and most closely-related to the ST313 isolates U7 and U9, from the UK [36].

**Fig 5:**
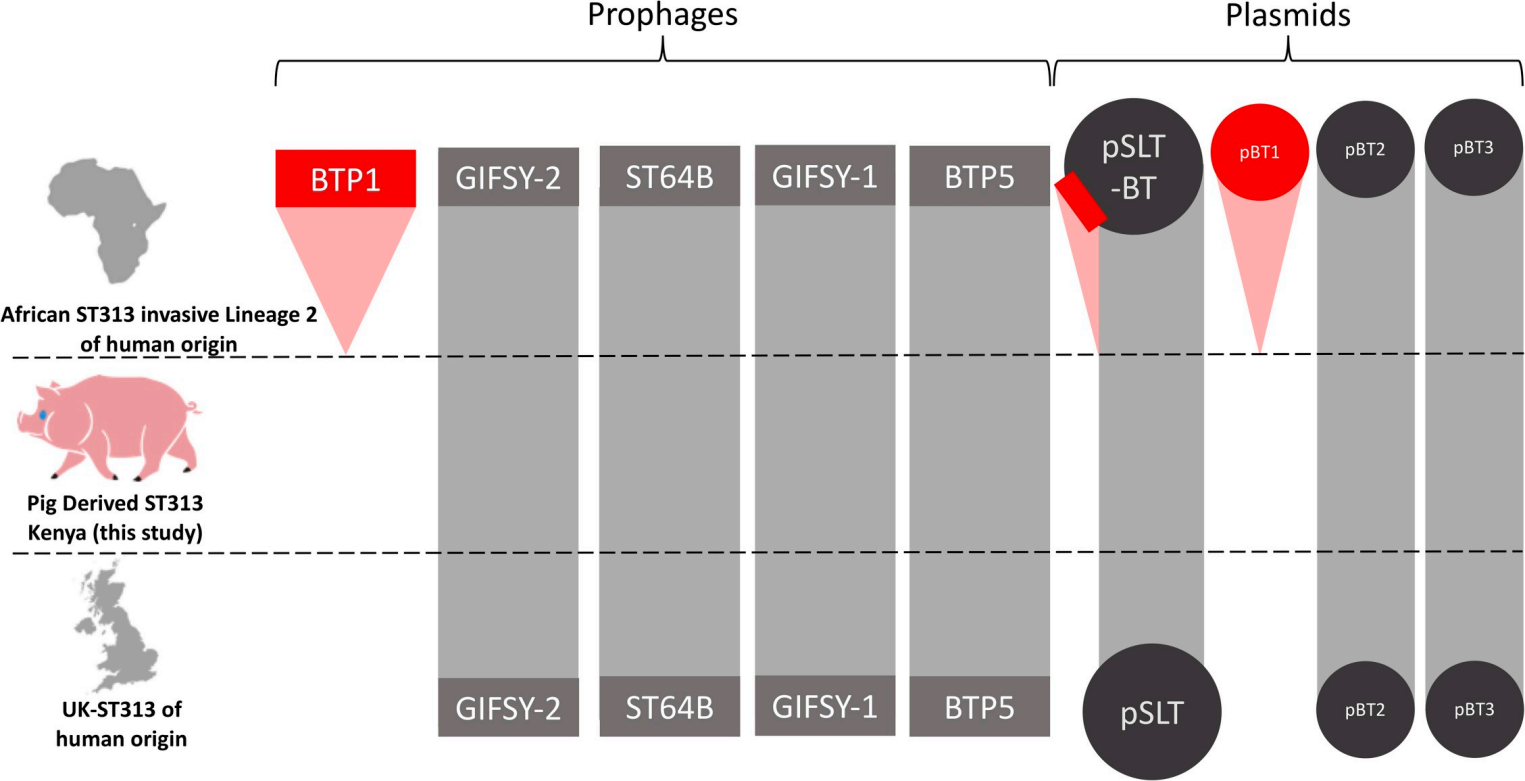
Comparison of the plasmid and prophage repertoires of *S*. Typhimurium ST313 variants. The presence and absence of *Salmonella* prophages BTP1, Gifsy-2 ST64B, Gifsy-1 and BTP5 and *Salmonella* plasmids pSLT-BT, pBT1, pBT2 and pBT3 are shown on three variants of *S*. Typhimurium ST313. Grey indicates similarity above 95% to African ST313 lineage 2 reference genome D23580. Red indicates absence compared to African ST313 lineage 2 reference genome D23580. The red rectangle on plasmid pSLT-BT represents the multidrug resistance cassette which is present in African ST313 lineage 2, but absent from UK-ST313 and the pig-derived ST313 isolates FD01844610 and FD01844641.

Accessory genome analysis revealed that the two pig-derived *S*. Typhimurium ST313 shared a similar prophage and plasmid repertoire to other ST313 isolates responsible for gastrointestinal disease in England and Wales. Importantly, neither belonged to African *S*. Typhimurium ST313 Lineage 2, which is currently causing the epidemic of iNTS in sSA (Fig 5). In terms of the African ST313 lineage 2 prophages, the two pig-derived ST313 carry Gifsy-2, ST64-B, Gifsy-1 and BTP5, but lack BTP1. In relation to the African ST313 lineage 2-associated plasmids [50] the two pig-derived ST313 isolates carry pBT2 and pBT3, but pBT1 is absent. The two pig-derived ST313 isolates carried the large *S*. Typhimurium virulence plasmid (pSLT), which did not contain the 10kb MDR gene cassette characteristic of African Lineage 2 ST313.

## Discussion

Our findings provide evidence of *Salmonella* carriage by pigs in Kenya and Malawi, and reveals the potential for pigs to act as a reservoir for human disease associated NTS serovars. We demonstrate that pigs in Kenya and Malawi carry and excrete a diverse range of *Salmonella* serovars to the environment, the majority of which (66%, 21/32) have previously caused gastroenteritis in Africa [39]. We found no evidence that pigs are a reservoir for the novel lineages of *S*. Typhimurium and *S*. Enteritidis particularly associated with invasive disease in sSA.

There is extensive knowledge of *Salmonella* in pigs in the USA and Europe [15–19], however, there is limited data from sSA [21]. As the African market for pork continues to grow, and because *Salmonella* is one of the most common causes of foodborne illness worldwide [37,51], increased knowledge of *Salmonella* excreted by pigs in sSA is required. In Europe and the USA, management strategies have been developed in pig farming to reduce the spread of this *Salmonella*, which have been most successful in Scandinavia [52]; locally relevant strategies are necessary for sSA.

Seven isolates of *S*. Typhimurium ST19 were detected from pigs slaughtered in Nairobi. Metadata indicates that three of the host pigs were reared within a five-kilometre radius of each other and differed by less than 5 core genome SNPs, raising the possibility that the ST19 strain had been transmitted between these pigs during rearing, transport or slaughter and could pose a threat to human health. The consequent opportunity for human exposure to zoonotic *Salmonella* at any of these stages raises serious public health concerns, and highlights the need for on-farm studies. There is clearly scope to better understand the transmission dynamics of such strains *in situ*.

Two isolates of particular relevance were *S*. Typhimurium ST313, a sequence type responsible for almost two thirds of iNTS cases in Malawi, and never previously found in food animals in Africa. Core gene phylogenetic analysis showed that the isolates from pigs in the Nairobi abattoir were related to a diverse group of ST313 isolates which currently cause gastrointestinal disease in humans in England and Wales [36] (Fig 4). Importantly from a public health perspective, the two pig-derived *S*. Typhimurium ST313 isolates were not closely related to lineages of ST313 associated with iNTS disease in Africa. We have not found that pigs are a reservoir for *Salmonella* strains which are strongly associated with *Salmonella* causing invasive disease in Africa, i.e. African ST313s.

*S*. Enteritids and *S*. Typhimurium are responsible for nearly 90% of all human NTS infections in sSA [4,53]. Within this study only a small number of *S*. Enteritidis and *S*. Typhimurium isolates have been detected. Therefore, only a limited number of the isolates detected are of critical importance to humans as a potential zoonoses.

The majority of pig-derived *Salmonella* isolates were susceptible to all antibiotics tested, and no isolates were classified as multidrug-resistant (resistant to three or more classes of antibiotics). One *gyrA* mutation was identified in a single pig-derived isolate indicating genotypic resistance to fluoroquinolones. Fluoroquinolone antibiotics are increasingly being used in African human clinics [54], but evidence from Nairobi suggests that fluoroquinolones are used less frequently to treat animals in the veterinary sector [55].

This study reports porcine reservoirs of zoonotic diarrhoeal-causing NTS serovars in sSA, and did not find evidence of pigs as a reservoir for lineages of ST313 associated with invasive disease. As *Salmonella* is one of the most common causes of foodborne illness worldwide [56] there is a need for coordinated national epidemiological surveillance programmes to monitor food borne pathogens in pork production in sSA, especially as this industry expands. Such information will facilitate the development of intervention strategies aimed at limiting the cases of human *Salmonella* disease linked to transmission of *Salmonella* spp. from pigs in sSA.

## Supporting information

S1 Fig Pairwise comparison of the genomes of the two *Salmonella* ST313 genomes isolated visualised using the Artemis Comparison Tool [44]. (PPTX)

S2 Fig Location of slaughter of pigs found to be carrying NTS isolates in each of the three study locations, correlated to the phylogenetic tree of NTS isolates. A = Complete sample sites, B = Busia, C = Chikwawa, Malawi, D = Nairobi Link to Microreact figure online: https://microreact.org/project/BJOPB1IQE.
(PPTX)

S1 Table Sampling strategy and description of rearing methods in each study site. (DOCX)

S2 Table Contextual metadata. This table displays metadata and accession numbers for previously published *S*. Typhimurium ST313.
(XLSX)

S3 Table Dataset Summary. A full list of the serovars of NTS which were identified in this study. Clade, total percentage of each serovar of the total detected and as well as the location in which the serovar was detected, are included.
(DOCX)

S4 Table Strain Metadata. This table displays the metadata and accession numbers for all samples in this study. Figshare link: https://figshare.com/s/522fe3568eff05324bd6
(XLSX)

## References

[1] Laupland KB, Schønheyder HC, Kennedy KJ, Lyytikäinen O, Valiquette L, Galbraith J, et al Salmonella enterica bacteraemia: a multi-national population-based cohort study. BMC Infect Dis 2010;10:95 10.1186/1471-2334-10-95 20398281 PMC2861061

[2] Feasey NA, Masesa C, Jassi C, Faragher EB, Mallewa J, Mallewa M, et al Three Epidemics of Invasive Multidrug-Resistant Salmonella Bloodstream Infection in Blantyre, Malawi, 1998–2014. Clinical Infectious Diseases: An Official Publication of the Infectious Diseases Society of America 2015;61 Suppl 4:S363–71. 10.1093/cid/civ691 26449953 PMC4596930

[3] Reddy EA, Shaw AV, Crump JA. Community-acquired bloodstream infections in Africa: a systematic review and meta-analysis. Lancet Infect Dis 2010;10:417–32. 10.1016/S1473-3099(10)70072-4 20510282 PMC3168734

[4] Uche IV, MacLennan CA, Saul A. A Systematic Review of the Incidence, Risk Factors and Case Fatality Rates of Invasive Nontyphoidal Salmonella (iNTS) Disease in Africa (1966 to 2014). PLoS Negl Trop Dis 2017;11 10.1371/journal.pntd.0005118. PMC5215826 28056035

[5] Marchello CS, Dale AP, Pisharody S, Rubach MP, Crump JA. A Systematic Review and Meta-analysis of the Prevalence of Community-Onset Bloodstream Infections among Hospitalized Patients in Africa and Asia. Antimicrob Agents Chemother 2019;64:e01974–19, /aac/64/1/AAC.01974-19.atom. 10.1128/AAC.01974-19 31636071 PMC7187598

[6] Gordon M, Banda H, Gondwe M, Gordon S, Boeree M, Walsh A, et al Non-typhoidal salmonella bacteraemia among HIV-infected Malawian adults: high mortality and frequent recrudescence. Aids 2002;16:1633–41. 10.1097/00002030-200208160-00009 12172085

[7] Scott JAG, Berkley JA, Mwangi I, Ochola L, Uyoga S, Macharia A, et al Relation between falciparum malaria and bacteraemia in Kenyan children: a population-based, case-control study and a longitudinal study. Lancet 2011;378:1316–23. 10.1016/S0140-6736(11)60888-X 21903251 PMC3192903

[8] Berkley JA, Lowe BS, Mwangi I, Williams T, Bauni E, Mwarumba S, et al Bacteremia among Children Admitted to a Rural Hospital in Kenya. Http://DxDoiOrg/101056/NEJMoa040275 2009 10.1056/NEJMoa040275. 15635111

[9] Kingsley RA, Msefula CL, Thomson NR, Kariuki S, Holt KE, Gordon MA, et al Epidemic multiple drug resistant Salmonella Typhimurium causing invasive disease in sub-Saharan Africa have a distinct genotype. Genome Res 2009;19:2279–87. 10.1101/gr.091017.109 19901036 PMC2792184

[10] Feasey NA, Hadfield J, Keddy KH, Dallman TJ, Jacobs J, Deng X, et al Distinct Salmonella Enteritidis lineages associated with enterocolitis in high-income settings and invasive disease in low-income settings. Nat Genet 2016;48:1211–7. 10.1038/ng.3644 27548315 PMC5047355

[11] Post AS, Diallo SN, Guiraud I, Lompo P, Tahita MC, Maltha J, et al Supporting evidence for a human reservoir of invasive non-Typhoidal Salmonella from household samples in Burkina Faso. PLOS Neglected Tropical Diseases 2019;13:e0007782 10.1371/journal.pntd.0007782 31609964 PMC6812844

[12] Okoro CK, Barquist L, Connor TR, Harris SR, Clare S, Stevens MP, et al Signatures of Adaptation in Human Invasive Salmonella Typhimurium ST313 Populations from Sub-Saharan Africa. PLOS Neglected Tropical Diseases 2015;9:e0003611 10.1371/journal.pntd.0003611 25803844 PMC4372345

[13] Nollet N, Maes D, Duchateau L, Hautekiet V, Houf K, Van Hoof J, et al Discrepancies between the isolation of Salmonella from mesenteric lymph nodes and the results of serological screening in slaughter pigs. Vet Res 2005;36:545–55. 10.1051/vetres:2005014 15955280

[14] Mirceta J, Petrovic J, Malesevic M, Blagojevic B, Antic D. Assessment of microbial carcass contamination of hunted wild boars. Eur J Wildl Res 2017;63:37 10.1007/s10344-017-1096-3.

[15] Bonardi S. Salmonella in the pork production chain and its impact on human health in the European Union. Epidemiology and Infection; Cambridge 2017;145:1513–26. http://dx.doi.org.liverpool.idm.oclc.org/10.1017/S095026881700036X. 10.1017/S095026881700036X PMC9203350 28241896

[16] Elnekave E, Hong S, Mather AE, Boxrud D, Taylor AJ, Lappi V, et al Salmonella enterica Serotype 4,[5],12:i:- in Swine in the United States Midwest: An Emerging Multidrug-Resistant Clade. Clin Infect Dis 2018;66:877–85. 10.1093/cid/cix909 29069323

[17] Zhang S, Li S, Gu W, den Bakker H, Boxrud D, Taylor A, et al Zoonotic Source Attribution of Salmonella enterica Serotype Typhimurium Using Genomic Surveillance Data, United States. Emerging Infect Dis 2019;25:82–91. 10.3201/eid2501.180835. PMC6302586 30561314

[18] Lewerin SS, Skog L, Frössling J, Wahlström H. Geographical distribution of salmonella infected pig, cattle and sheep herds in Sweden 1993–2010. Acta Vet Scand 2011;53:51 10.1186/1751-0147-53-51 21975258 PMC3198682

[19] Baptista FM, Dahl J, Nielsen LR. Factors influencing Salmonella carcass prevalence in Danish pig abattoirs. Prev Vet Med 2010;95:231–8. 10.1016/j.prevetmed.2010.04.007 20537741

[20] Pires S, de Knegt L, Hald T. Estimation of the relative contribution of different food and animal sources to human Salmonella infections in the European Union 2011.

[21] Afema JA, Byarugaba DK, Shah DH, Atukwase E, Nambi M, Sischo WM. Potential Sources and Transmission of Salmonella and Antimicrobial Resistance in Kampala, Uganda. PLOS ONE 2016;11:e0152130 10.1371/journal.pone.0152130 26999788 PMC4801205

[22] Acosta A. Market perspectives for the livestock sector in Africa: a vector autoregressive approach, Addis Ababa, Ethiopia: 2016, p. 1–9.

[23] Index Box Marketing and Consulting. Africa- Pork (Meat of Swine)- Market analysis, forecast, size, trends and insights 2019.

[24] Thomas LF, de Glanville WA, Cook EA, Fèvre EM. The spatial ecology of free-ranging domestic pigs (Sus scrofa) in western Kenya. BMC Vet Res 2013;9:46 10.1186/1746-6148-9-46 23497587 PMC3637381

[25] An accessible, efficient and global approach for the large-scale sequencing of bacterial genomes | bioRxiv n.d. https://www.biorxiv.org/content/10.1101/2020.07.22.200840v1 (accessed September 10, 2020). 10.1186/s13059-021-02536-3 PMC8690886 34930397

[26] Wood DE, Salzberg SL. Kraken: ultrafast metagenomic sequence classification using exact alignments. Genome Biology 2014;15:R46 10.1186/gb-2014-15-3-r46 24580807 PMC4053813

[27] Bolger AM, Lohse M, Usadel B. Trimmomatic: a flexible trimmer for Illumina sequence data. Bioinformatics (Oxford, England) 2014;30:2114–20. 10.1093/bioinformatics/btu170 24695404 PMC4103590

[28] Wick RR, Judd LM, Gorrie CL, Holt KE. Unicycler: Resolving bacterial genome assemblies from short and long sequencing reads. PLoS Computational Biology 2017;13:e1005595 10.1371/journal.pcbi.1005595 28594827 PMC5481147

[29] Alikhan N-F, Zhou Z, Sergeant MJ, Achtman M. A genomic overview of the population structure of Salmonella. PLoS Genetics 2018;14:e1007261 10.1371/journal.pgen.1007261 29621240 PMC5886390

[30] Seemann T. Prokka: rapid prokaryotic genome annotation. Bioinformatics (Oxford, England) 2014;30:2068–9. 10.1093/bioinformatics/btu153 24642063

[31] Yoshida CE, Kruczkiewicz P, Laing CR, Lingohr EJ, Gannon VPJ, Nash JHE, et al The Salmonella In Silico Typing Resource (SISTR): An Open Web-Accessible Tool for Rapidly Typing and Subtyping Draft Salmonella Genome Assemblies. PloS One 2016;11:e0147101 10.1371/journal.pone.0147101 26800248 PMC4723315

[32] Larsen MV, Cosentino S, Rasmussen S, Friis C, Hasman H, Marvig RL, et al Multilocus Sequence Typing of Total-Genome-Sequenced Bacteria. J Clin Microbiol 2012;50:1355–61. 10.1128/JCM.06094-11 22238442 PMC3318499

[33] Page AJ, Cummins CA, Hunt M, Wong VK, Reuter S, Holden MTG, et al Roary: rapid large-scale prokaryote pan genome analysis. Bioinformatics (Oxford, England) 2015;31:3691–3. 10.1093/bioinformatics/btv421 26198102 PMC4817141

[34] Stamatakis A. RAxML version 8: a tool for phylogenetic analysis and post-analysis of large phylogenies. Bioinformatics (Oxford, England) 2014;30:1312–3. 10.1093/bioinformatics/btu033 24451623 PMC3998144

[35] Letunic I, Bork P. Interactive tree of life (iTOL) v3: an online tool for the display and annotation of phylogenetic and other trees. Nucleic Acids Research 2016;44:W242–5. 10.1093/nar/gkw290 27095192 PMC4987883

[36] Ashton PM, Owen S V, Kaindama L, Rowe WPM, Lane CR, Larkin L, et al Public health surveillance in the UK revolutionises our understanding of the invasive Salmonella Typhimurium epidemic in Africa. Genome Medicine 2017;9:92 10.1186/s13073-017-0480-7 29084588 PMC5663059

[37] Tran-Dien A, Hello SL, Bouchier C, Weill F-X. Early transmissible ampicillin resistance in zoonotic Salmonella enterica serotype Typhimurium in the late 1950s: a retrospective, whole-genome sequencing study. The Lancet Infectious Diseases 2018;18:207–14. 10.1016/S1473-3099(17)30705-3 29198740

[38] Almeida F, Seribelli AA, da Silva P, Medeiros MIC, Dos Prazeres Rodrigues D, Moreira CG, et al Multilocus sequence typing of Salmonella Typhimurium reveals the presence of the highly invasive ST313 in Brazil. Infection, Genetics and Evolution: Journal of Molecular Epidemiology and Evolutionary Genetics in Infectious Diseases 2017;51:41–4. 10.1016/j.meegid.2017.03.009 28288927

[39] Msefula CL, Kingsley RA, Gordon MA, Molyneux E, Molyneux ME, MacLennan CA, et al Genotypic Homogeneity of Multidrug Resistant S. Typhimurium Infecting Distinct Adult and Childhood Susceptibility Groups in Blantyre, Malawi. PLOS ONE 2012;7:e42085 10.1371/journal.pone.0042085 22848711 PMC3407126

[40] Okoro CK, Kingsley RA, Connor TR, Harris SR, Parry CM, Al-Mashhadani MN, et al Intracontinental spread of human invasive Salmonella Typhimurium pathovariants in sub-Saharan Africa. Nature Genetics 2012;44:1215–21. 10.1038/ng.2423 23023330 PMC3491877

[41] Page AJ, Taylor B, Delaney AJ, Soares J, Seemann T, Keane JA, et al SNP-sites: rapid efficient extraction of SNPs from multi-FASTA alignments. Microbial Genomics 2016;2:e000056 10.1099/mgen.0.000056 28348851 PMC5320690

[42] Inouye M, Dashnow H, Raven L-A, Schultz MB, Pope BJ, Tomita T, et al SRST2: Rapid genomic surveillance for public health and hospital microbiology labs. Genome Medicine 2014;6:90 10.1186/s13073-014-0090-6 25422674 PMC4237778

[43] Assefa S, Keane TM, Otto TD, Newbold C, Berriman M. ABACAS: algorithm-based automatic contiguation of assembled sequences. Bioinformatics (Oxford, England) 2009;25:1968–9. 10.1093/bioinformatics/btp347 19497936 PMC2712343

[44] Carver TJ, Rutherford KM, Berriman M, Rajandream M-A, Barrell BG, Parkhill J. ACT: the Artemis Comparison Tool. Bioinformatics (Oxford, England) 2005;21:3422–3423. 10.1093/bioinformatics/bti553 15976072

[45] Zankari E, Hasman H, Cosentino S, Vestergaard M, Rasmussen S, Lund O, et al Identification of acquired antimicrobial resistance genes. J Antimicrob Chemother 2012;67:2640–4. 10.1093/jac/dks261 22782487 PMC3468078

[46] Zankari E, Allesøe R, Joensen KG, Cavaco LM, Lund O, Aarestrup FM. PointFinder: a novel web tool for WGS-based detection of antimicrobial resistance associated with chromosomal point mutations in bacterial pathogens. Journal of Antimicrobial Chemotherapy 2017;72:2764–8. 10.1093/jac/dkx217 29091202 PMC5890747

[47] Matuschek E, Brown DFJ, Kahlmeter G. Development of the EUCAST disk diffusion antimicrobial susceptibility testing method and its implementation in routine microbiology laboratories. Clinical Microbiology and Infection: The Official Publication of the European Society of Clinical Microbiology and Infectious Diseases 2014;20:O255–66. 10.1111/1469-0691.12373 24131428

[48] Andrews JM, Howe RA. BSAC standardized disc susceptibility testing method (version 10). The Journal of Antimicrobial Chemotherapy 2011;66:2726–57. 10.1093/jac/dkr359 21921076

[49] den Bakker HC, Moreno Switt AI, Govoni G, Cummings CA, Ranieri ML, Degoricija L, et al Genome sequencing reveals diversification of virulence factor content and possible host adaptation in distinct subpopulations of Salmonella enterica. BMC Genomics 2011;12:425 10.1186/1471-2164-12-425 21859443 PMC3176500

[50] Canals R, Hammarlöf DL, Kröger C, Owen SV, Fong WY, Lacharme-Lora L, et al Adding function to the genome of African Salmonella Typhimurium ST313 strain D23580. PLOS Biology 2019;17:e3000059 10.1371/journal.pbio.3000059 30645593 PMC6333337

[51] WHO Estimates of the Global Burden of Foodborne Diseases n.d.

[52] Helwigh B, Christensen J, Müller L, Munck N, de Knegt L, Hald T, et al Annual Report on Zoonoses in Denmark 2015. 2016 10.13140/RG.2.2.22803.68647.

[53] Phoba M-F, Barbé B, Ley B, Van Puyvelde S, Post A, Mattheus W, et al High genetic similarity between non-typhoidal Salmonella isolated from paired blood and stool samples of children in the Democratic Republic of the Congo. PLoS Negl Trop Dis 2020;14 10.1371/journal.pntd.0008377 32614856 PMC7331982

[54] Chattaway MA, Aboderin AO, Fashae K, Okoro CK, Opintan JA, Okeke IN. Fluoroquinolone-Resistant Enteric Bacteria in Sub-Saharan Africa: Clones, Implications and Research Needs. Front Microbiol 2016;7 10.3389/fmicb.2016.00007 27148238 PMC4841292

[55] Muloi D, Fèvre EM, Bettridge J, Rono R, Ong’are D, Hassell JM, et al A cross-sectional survey of practices and knowledge among antibiotic retailers in Nairobi, Kenya. J Glob Health n.d.;9 10.7189/jogh.09.020412 31489183 PMC6708591

[56] Majowicz SE, Musto J, Scallan E, Angulo FJ, Kirk M, O’Brien SJ, et al The global burden of nontyphoidal Salmonella gastroenteritis. Clinical Infectious Diseases: An Official Publication of the Infectious Diseases Society of America 2010;50:882–9. 10.1086/650733 20158401

[57] Kröger C, Dillon SC, Cameron ADS, Papenfort K, Sivasankaran SK, Hokamp K, et al The transcriptional landscape and small RNAs of Salmonella enterica serovar Typhimurium. PNAS 2012;109:E1277–86. 10.1073/pnas.1201061109. 22538806 PMC3356629

