# Supplementary figures and images for: Salmonella identified in pigs in Kenya and Malawi reveals the potential for zoonotic transmission in emerging pork markets
Source: PLoS Negl Trop Dis. 2020 Nov 24;14(11):e0008796. doi: 10.1371/journal.pntd.0008796 (PMC7748489; doi:10.1371/journal.pntd.0008796)

## Slide 1
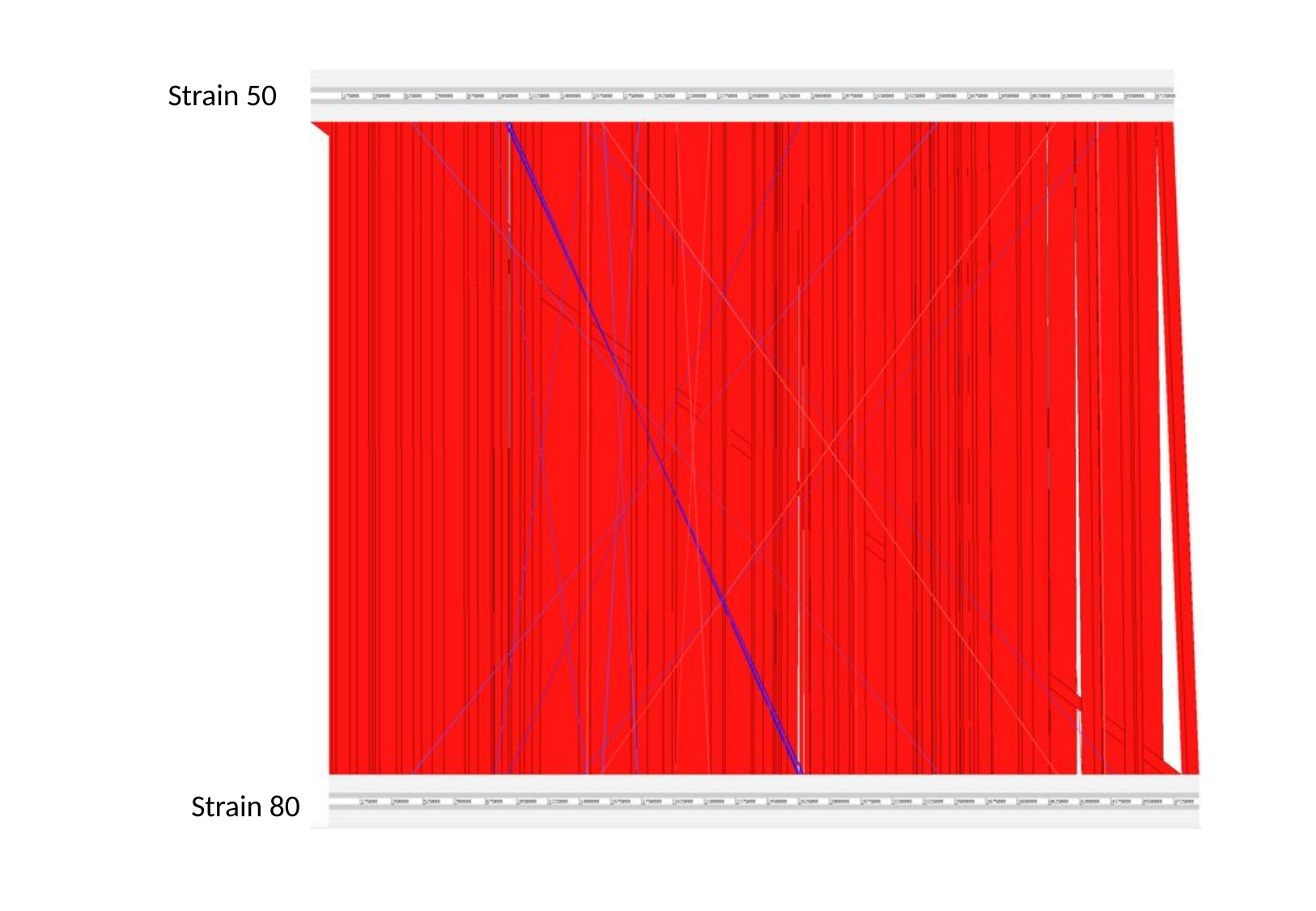

Strain 50
Strain 80

Supplement: S1 Fig — (PPTX) [file pntd.0008796.s001.pptx]
