# Supplementary material for: Salmonella identified in pigs in Kenya and Malawi reveals the potential for zoonotic transmission in emerging pork markets
Source: PLoS Negl Trop Dis. 2020 Nov 24;14(11):e0008796. doi: 10.1371/journal.pntd.0008796 (PMC7748489; doi:10.1371/journal.pntd.0008796)
Supplement: S2 Fig — A = Complete sample sites, B = Busia, C = Chikwawa, Malawi, D = Nairobi Link to Microreact figure online: https://microreact.org/project/BJOPB1IQE. (PPTX) [file pntd.0008796.s002.pptx]

## Slide 1
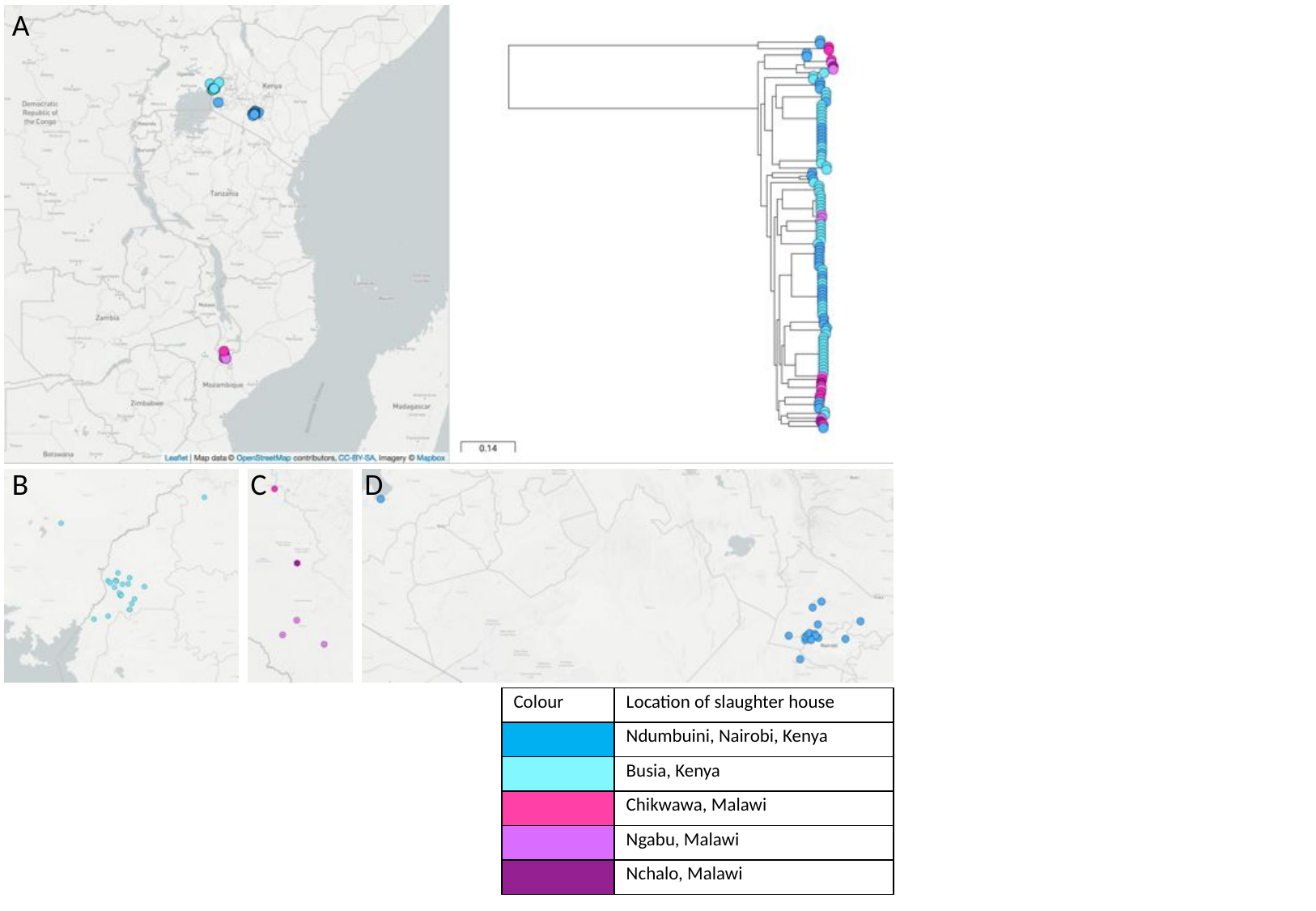

A
B
C
D
| Colour | Location of slaughter house |
| --- | --- |
| | Ndumbuini, Nairobi, Kenya |
| | Busia, Kenya |
| | Chikwawa, Malawi |
| | Ngabu, Malawi |
| | Nchalo, Malawi |
