# Supplementary material for: Salmonella identified in pigs in Kenya and Malawi reveals the potential for zoonotic transmission in emerging pork markets
Source: PLoS Negl Trop Dis. 2020 Nov 24;14(11):e0008796. doi: 10.1371/journal.pntd.0008796 (PMC7748489; doi:10.1371/journal.pntd.0008796)
Supplement: S1 Table — (DOCX) [file pntd.0008796.s003.docx]

**S1 Table: Sampling strategy and description of rearing methods in each study site**

| **Location** | **Sampling Details** | **Rearing methods in local area** |
| --- | --- | --- |
| Busia, Kenya | Samples collected from 7 slaughter slabs all located within 1-hour drive of Busia town. | Traditionally reared via low-intensity methods, free-roaming and scavenging around the homesteads and villages to gain access to food [24]. |
| Nairobi, Kenya | Samples collected from swine slaughterhouse on the peri-urban fringe of the city. The majority of these pigs originate from surrounding rural and peri-urban areas, and some originate from further afield in Kenya. | Often reared in a more commercial, intensive environment than found in Busia or Chikwawa. |
| Chikwawa, Malawi | Samples collected from pork butcheries, to which slaughtermen brought the MLN and faecal samples specifically for the study. Slaughter men collected samples within 1 hour of death; slaughter normally having occurred in the yard of the pig’s owner. Sample collection took place from butcheries located in three towns in the Chikwawa District, namely Chikwawa, Ngabu and Nchalo. |  |
