# Supplementary material for: Salmonella identified in pigs in Kenya and Malawi reveals the potential for zoonotic transmission in emerging pork markets
Source: PLoS Negl Trop Dis. 2020 Nov 24;14(11):e0008796. doi: 10.1371/journal.pntd.0008796 (PMC7748489; doi:10.1371/journal.pntd.0008796)
Supplement: S3 Table — A full list of the serovars of NTS which were identified in this study. Clade, total percentage of each serovar of the total detected and as well as the location in which the serovar was detected, are included. (DOCX) [file pntd.0008796.s005.docx]

**S3 Table: Dataset Summary**

A full list of the serovars of NTS which were identified in this study. Clade, total percentage of each serovar of the total detected and as well as the location in which the serovar was detected, are included.

| **Clade** | **Total number of non-typhoidal Salmonella isolates (n=121) (percentage in brackets)** | **Serovars** | **Number of isolates** | **Location** |
| --- | --- | --- | --- | --- |
| *S. salamae* | 4 (3.4) | II 1,4,12,27:e,n,x:e,n,x  II42:r:- | 2  2 | Chikwawa  Nairobi |
| *S. enterica* misc. | 7 (5.9) | Jangwani  Johannesburg  Mbandaka  Oranienburg | 2  2  2  1 | Chikwawa  Chikwawa  Nairobi  Busia |
| *S. enterica* clade B | 29 (23.5) | Fulica  Hadar  Kiambu  Offa  Stanleyville | 16  4  2  3  4 | Nairobi + Busia  Nairobi + Busia  Nairobi  Busia  Busia |
| *S. enterica* clade A | 79 (65.5) | Aberdeen  Amager  Anatum  Baildon  Bovismorbificans  Braenderup  Concord  Enteritidis  Fillmore  Guildford  Heidelberg  Hull  I41:b:-  Infantis  Kottbus  Muenchen  Newport  Typhimurium  Uganda  Virchow  Zanzibar | 1  1  2  4  3  3  1  1  1  6  15  1  2  1  1  3  15  8  6  2  2 | Busia  Chikwawa  Nairobi+Chikwawa  Chikwawa  Busia  Nairobi  Chikwawa  Busia  Chikwawa  Busia  Nairobi + Busia  Chikwawa  Chikwawa  Nairobi  Chikwawa  Nairobi  Nairobi + Busia  Nairobi  Busia  Nairobi  Chikwawa |
| Unknown | 2 (1.7) | Unknown | 2 | Busia |
